# Deep learning‐based 3D dose reconstruction for intensity modulated radiation therapy using electronic portal imaging devices

**DOI:** 10.1002/acm2.70328

**Published:** 2025-11-05

**Authors:** Dong Yang, Jiawei Fan, Yu Wenliang, Jiazhou Wang, Zihan Sun, Weigang Hu

**Affiliations:** ^1^ Department of Radiation Oncology Fudan University Shanghai Cancer Center; Department of Oncology, Shanghai Medical College Fudan University Shanghai China; ^2^ Department of Radiation Oncology Quzhou People’ s Hospital The Quzhou Affiliated Hospital of Wenzhou Medical University Quzhou China; ^3^ Department of Radiation Oncology The first affiliated hospital Zhejiang University school of medicine Hangzhou China

**Keywords:** deep learning (DL), dose reconstruction, electronic portal imaging device (EPID), patient specific quality assurance, transit dosimetry

## Abstract

**Background and purpose:**

Electronic portal imaging devices (EPIDs) have been used as an in vivo dosimetry method to identify treatment discrepancies by comparing planned and predicted 3D dose. The purpose of this study was to develop and evaluate a deep learning‐based framework for reconstructing three‐dimensional (3D) patient dose distributions directly from electronic portal imaging device (EPID) images and planning CT, without relying on Monte Carlo simulation or conventional back‐projection methods.

**Materials and methods:**

A Res‐UNet architecture was trained using 512 beam fields from 60 head and neck IMRT patients to predict beam‐specific dose slices from corresponding EPID images and CT data. For each beam, predicted dose slices were assembled into a 3D volume and summed across all beams to reconstruct the full patient dose. The model was evaluated on 10 independent cases using voxel‐wise mean absolute error (MAE), mean squared error (MSE), slice‐wise dose difference, 3D gamma analysis (global normalization, 10% low‐dose threshold) with criteria of 3%/3 mm, and dose‐volume histogram (DVH) comparisons. Statistical significance was assessed using the Wilcoxon signed‐rank test.

**Results:**

The model achieved a mean voxel‐wise MAE of 0.163 Gy, with over 95% of voxels with MAE < 2 Gy. The average slice‐level dose difference was 1.186 Gy, with localized underestimation observed in high‐dose gradient regions. The overall global 3D gamma passing rates were 90.67% for the 3%/3 mm criterion. DVH comparisons showed no statistically significant differences for most targets and organs‐at‐risk, except for D_2%_ of the left lens (*p* = 0.020) and *D*_mean_ of the parotid glands (*p* = 0.012), which demonstrated statistically significant differences. The average reconstruction time was 15 s per beam.

**Conclusion:**

The proposed framework demonstrates the feasibility of fast and patient‐specific 3D dose reconstruction using EPID and CT data without complex physical modeling. Under the predefined acceptance criterion (≥95% of voxels with MAE < 2 Gy), 7 out of 10 test cases met the standard. Although high‐gradient regions remain challenging and gamma passing rates for the 3%/3 mm criterion were slightly lower than in some prior studies, the method offers sufficient accuracy for many quality assurance applications and provides a foundation for future integration into online adaptive radiotherapy workflows.

## 1 INTRODUCTION

Accurate dose verification during radiotherapy is essential for ensuring treatment quality and patient safety. In vivo dosimetry methods provide direct information on the actual dose delivered to the patient, complementing conventional pre‐treatment quality assurance (QA) procedures that may not detect anatomical changes or delivery errors during treatment.^1^, ^2^, ^3^, ^4^

Among in vivo dosimetry techniques, electronic portal imaging devices (EPIDs) have gained increasing clinical attention due to their high spatial resolution, dose linearity, and ease of use.^5^, ^6^, ^7^, ^8^ Traditionally used for patient setup verification, EPIDs have also been applied to transit dosimetry by recording x‐ray fluence on a pixel‐based plane.^9^, ^10^, ^11^ EPID‐based dose reconstruction methods are typically classified as either forward or back‐projection systems.^12^, ^13^, ^14^, ^15^ Forward projection methods compare measured EPID images with predicted portal doses calculated from treatment plans, often using Monte Carlo or analytical models.^16^, ^17^, ^18^ Back‐projection approaches estimate the incident fluence on the EPID and reconstruct the internal dose by modeling the patient geometry and energy deposition.^19^, ^20^, ^21^, ^22^, ^23^ Although back‐projection enables 3D dose reconstruction and dose‐volume histogram (DVH)‐based evaluation, it often requires complex EPID response modeling, patient scatter correction, and computationally intensive simulation workflows.^24^, ^25^, ^26^, ^27^, ^28^, ^29^, ^30^, ^31^, ^32^, ^33^

To address these limitations, deep learning (DL) methods have been proposed to directly learn the mapping between EPID images and patient dose distributions. Prior studies have demonstrated DL models for predicting gamma passing rates,^34^, ^35^ detecting delivery errors,^36^, ^37^ and reconstructing planar or volumetric doses using EPID data.^38^, ^39^, ^40^ However, these methods often require Monte Carlo pre‐processing, coarse dose inputs, or 2D slice stacking with interpolation, which can limit practical clinical adoption.

In this study, we present a deep learning framework based on a Res‐UNet architecture for direct 3D patient dose reconstruction using EPID images and planning CT scans, without Monte Carlo simulation. Our approach is designed for head and neck IMRT cases, reconstructing dose distributions from individual beam projections, and aims to evaluate the feasibility, accuracy, and efficiency of this method for patient‐specific transit dosimetry.

## 2 MATERIALS AND METHODS

### 2.1 Patient data and imaging acquisition

This retrospective study included 70 patients with head and neck cancer who underwent fixed‐beam IMRT treatment (6 MV, 600 MU/min) at our institution. Treatments were delivered using the UIH CT‐Linac 506c system (United Imaging Healthcare, Shanghai, China), equipped with a 16‐slice helical CT scanner and an amorphous silicon EPID (XRD1642, Varex Imaging, UT, USA). Ethics approval was obtained from our institutional review board.

The dataset included a total of 512 beams from 60 patients for model training and validation (5‐fold cross‐validation, split by patient to avoid data leakage), and 90 beams from 10 completely unseen patients for testing. To enhance model robustness, we applied geometric data augmentation (translation and flipping along x/y axes) to 25% of the dataset. Augmentation was applied simultaneously to CT, EPID, and dose images to ensure spatial correspondence.

CT images were acquired at 150 kV, with 3–5 mm slice thickness and a 512 × 512 in‐plane matrix. All CTs were resampled to an isotropic voxel size of 1.25 × 1.25 × 5 mm^3^ using cubic interpolation and padded symmetrically in the x‐y plane to generate 512 × 512 axial slices centered at the isocenter. For each beam, 120 slices were selected along the beam axis, spanning ± 300 mm longitudinally with a slice thickness of 5 mm. Both the full planning CT and the current slice to be reconstructed were input into the model, capturing both local anatomical detail and global anatomical context.

EPID images were not measured during treatment, but rather simulated via a Monte Carlo‐based engine embedded in the UIH treatment planning system (TPS), using photon phase space files and calibrated using dose‐per‐MU response mapping.^41^, ^42^ These simulated portal dose images (512 × 512 matrix, 0.8 mm binned pixels) represent the expected dose at the EPID plane under ideal delivery conditions. This simulation‐based approach ensures reproducibility and removes measurement noise, while approximating realistic EPID responses under TPS‐planned parameters. The source‐to‐image distance (SID) was 145 cm, resulting in an in‐plane resolution of 0.55 mm at the isocenter.

Planned 3D dose distributions exported from TPS were used as the ground truth. These were beam‐separated and aligned to the processed CT slices. For each beam, dose slices along the beam path were extracted from the TPS dose and paired with corresponding CT and EPID data for supervised learning. This provided a consistent training target aligned with the anatomy and projection geometry.

### 2.2 EPID‐to‐patient dose modeling

During radiotherapy delivery, EPID images capture the exit radiation fluence transmitted through the patient, encoding information about the anatomical attenuation and beam modulation along the incident path. In this study, the EPID image for each beam was treated as a 2D projection of accumulated beam‐specific energy deposition, corresponding to a cumulative shadow of patient anatomy and beam fluence modulated by treatment plan parameters.

To reconstruct 3D dose from 2D EPID images, a depth‐wise mapping framework was adopted. The patient body was sampled slice‐by‐slice along the beam axis (source to EPID direction), and for each depth slice, the corresponding portion of the EPID image was geometrically resized to reflect perspective distortion at that depth. Specifically, as the slice‐to‐source distance increases, the projected field of view on the EPID expands due to beam divergence. Therefore, the EPID image was rescaled for each reconstruction slice based on the source‐to‐slice and source‐to‐EPID distances, following the geometric magnification factor:

(1) $$scale\;factor=\frac{d_{slice}}{d_{EPID}}\;$$

In addition, a depth‐dependent correction was applied to account for the inverse square law governing radiation intensity, approximating the relationship between signal at the detector and dose at depth. The overall signal‐dose relationship was modeled as:

(2) $$D_{res}\;\left(x,y,d\right)=\left(S_{ori}\left(x,y\right)\cdot {\left(\frac{d}{d_{EPID}}\right)}^{-2}\right)\;⊗K_{\left(x,y,d\right)}$$

where $D_{res}$ is the reconstructed dose at point (x,y,d) inside the patient, $S_{ori}\left(x,y\right)$ is the signal at pixel (x,y), d is the source‐to‐slice distance, $d_{EPID}$ is the source‐to‐EPID distance, and $K_{\left(x,y,d\right)}$ encapsulates the complex attenuation, scatter, and anatomical modulation effects along the beam path. Rather than modeling these factors explicitly via analytical kernels or Monte Carlo simulation, we relied on the deep learning model to implicitly learn the transformation from EPID signal and CT anatomy to the corresponding beam‐specific dose distribution. The network was thus trained to approximate this depth‐aware mapping function slice‐by‐slice, using TPS dose as supervision. This formulation enables efficient, per‐beam 3D dose reconstruction while preserving physical interpretability and compatibility with conventional radiation geometry.

### 2.3 Deep learning model

A deep convolutional neural network based on a Res‐UNet architecture was developed to perform 3D patient dose reconstruction from portal images and planning CT data. The model extends the conventional U‐Net architecture by integrating residual connections to improve gradient flow and representation capacity. It consists of separate encoding and decoding pathways with long skip connections that preserve high‐resolution features across layers, enhancing structural consistency in the predicted dose.

**FIGURE 1:**
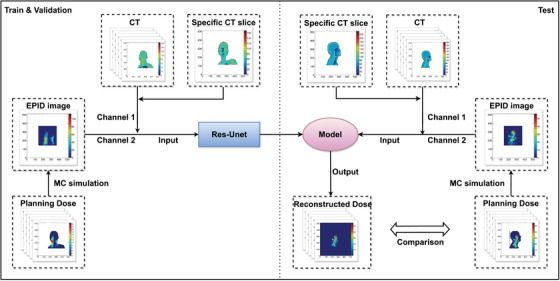
Overall workflow of the EPID‐based 3D patient dose reconstruction using deep learning.

**FIGURE 2:**
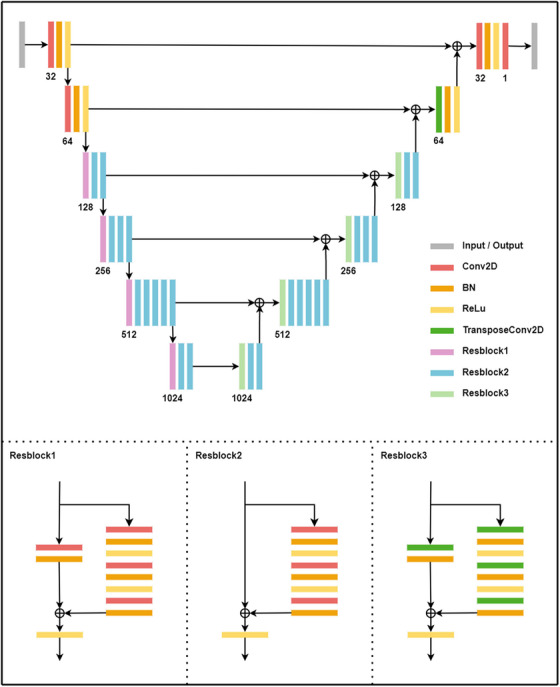
Architecture of the Res‐Unet deep learning model.

Each encoding block contains two 2D convolutional layers with 7 × 7 and 3 × 3 kernels, using specific stride settings to perform downsampling. At the bottleneck, a Conv2D layer is used to reduce the spatial resolution to the lowest feature level. In the decoding pathway, transposed convolutional layers with 3 × 3 kernels and a stride of 2 are used to upsample the feature maps. Residual blocks are incorporated throughout to improve training stability. The final output is a single‐channel 512 × 512 dose slice corresponding to the input reconstruction plane. The overall framework and model architecture are illustrated in Figures 1 and 2.

The network accepted two input channels: (1) a composite image combining the full planning CT and the current target CT slice to provide both anatomical context and local detail; and (2) a depth‐adjusted and geometrically scaled EPID image corresponding to the same beam angle and slice depth. No normalization was applied to CT or EPID inputs in order to preserve their physical meaning (HU values and absolute dose‐related signals), allowing the model to learn a patient‐specific mapping in absolute dose units. Dose values were retained in their original physical units as exported from the TPS, ensuring direct clinical interpretability.

The model was trained using a mean absolute error (MAE) loss function. Optimization was performed using the Adam optimizer with parameters *β*_1_ = 0.5 and *β*_2_ = 0.999, and an initial learning rate of 1 × 10^−5^ The batch size was set to 2. The model was implemented using PyTorch and trained for 5 epochs on an NVIDIA RTX 2080Ti GPU (12 GB). The best‐performing checkpoint was selected based on validation loss.

### 2.4 Evaluation and statistical analysis

To evaluate model performance, we reconstructed the 3D dose distributions for 10 independent head and neck IMRT test cases. The predicted beam doses were first assembled from slice‐wise outputs and then rotated and summed across all beams to obtain the final cumulative dose volume. Reconstruction accuracy was assessed by comparing the predicted dose with the corresponding TPS‐calculated dose using multiple quantitative metrics. These included voxel‐wise MAE and mean squared error (MSE), average dose differences per axial slice, and voxel error histograms within the body contour.

In addition, DVHs were generated for the gross tumor volume (GTV), planning target volume (PTV), and organs at risk (OARs). Clinically relevant DVH indices were extracted, including *D*_95%_ for the GTV and PTV, *D*_2%_ for critical structures (brainstem, spinal cord, optic nerves, chiasm, temporal lobes, and lenses), and *D*_mean_ for the parotid glands. Differences between the reconstructed and planned dose indices were analyzed using the Wilcoxon signed‐rank test, with statistical significance defined as *p* < 0.05.

The reconstructed doses were compared with the corresponding TPS‐calculated doses using a 3D gamma analysis. Gamma passing rates were computed with global dose normalization (maximum planned dose as reference). Criteria of 3%/3 mm were applied with a 10% low‐dose threshold to exclude low‐dose regions from the analysis. In addition, we defined a reconstruction as clinically acceptable if ≥95% of evaluated voxels had an absolute dose error < 2 Gy. This threshold was chosen because a 2 Gy deviation is generally within standard clinical tolerance levels for both targets and OARs in conventional fractionation.

To investigate whether plan modulation influences reconstruction accuracy, we quantified modulation complexity using the average beam MU normalized by the prescription dose. For each of the 10 independent test patients, the mean modulation number (± SD across all beams) and the patient‐level mean slice‐wise MAE were calculated. The association between modulation number and MAE was then evaluated using Spearman's rank correlation coefficient to capture monotonic trends. All correlation analyses were performed at the patient level to avoid pseudo‐replication, and statistical significance was set at *p* < 0.05.

### 2.5 Use of generative AI in manuscript preparation

AI tools (ChatGPT, OpenAI) were used to assist with language refinement and grammar editing. All scientific content, data interpretation, and final revisions were performed and verified by the authors. The authors accept full responsibility for the integrity and accuracy of the manuscript.

## 3 RESULTS

The proposed deep learning‐based framework demonstrated the feasibility of reconstructing 3D patient dose distributions directly from portal images and CT data for head and neck IMRT. The average computation time for dose reconstruction was approximately 15 s per beam.

### 3.1 Dose distribution accuracy

**FIGURE 3:**
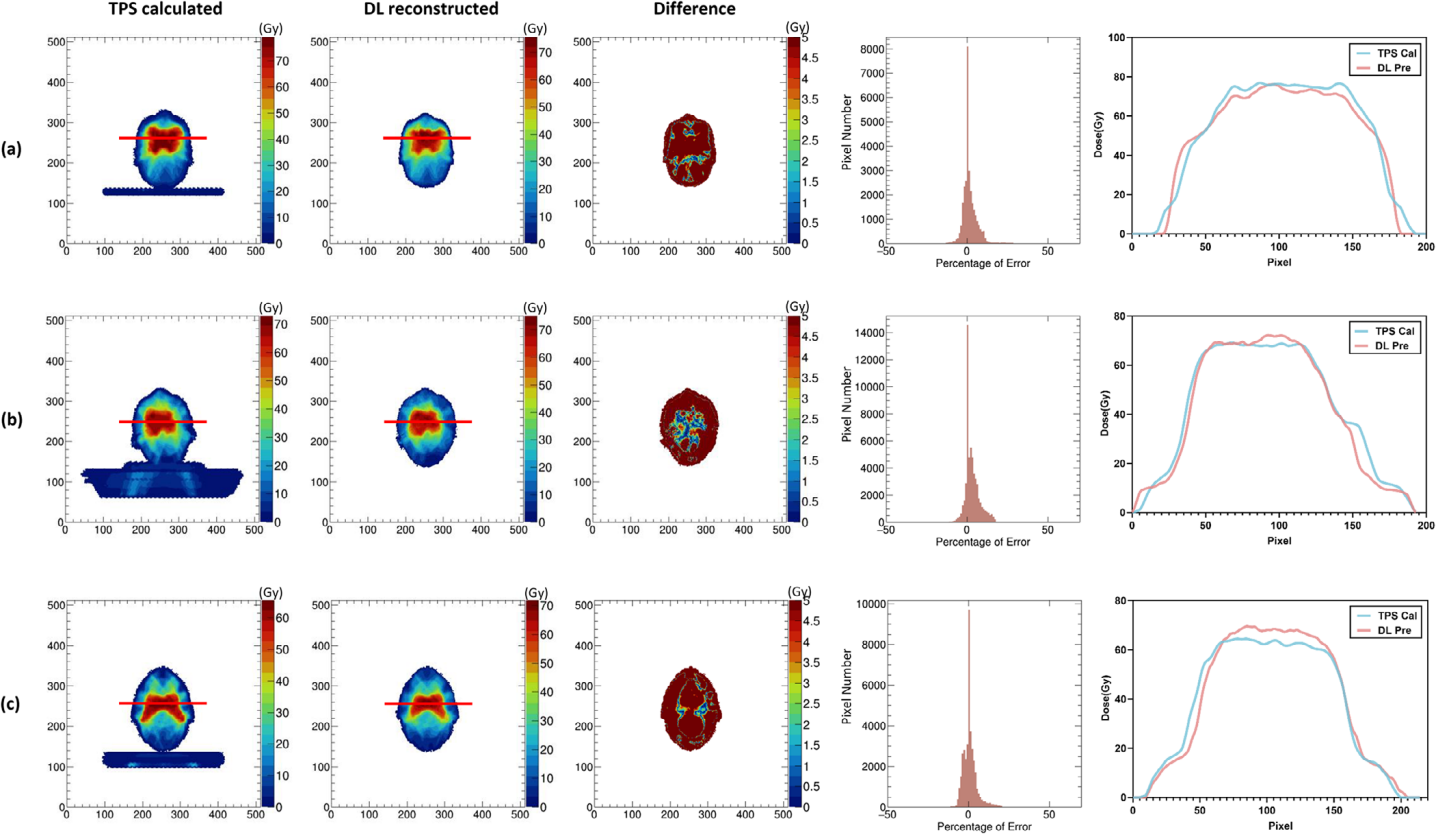
The individual beam dose distributions of three different gantry angles for three test cases calculated by the TPS (left column), reconstructed by DL model (middle column), and pixel‐wise dose difference between them (right column), respectively in (a–c).

Figure 3 illustrates per‐beam dose distributions at three different gantry angles for representative test cases, comparing the TPS‐calculated dose, the reconstructed dose, and the corresponding pixel‐wise differences. The reconstructed dose distributions were visually consistent with the TPS reference, with most discrepancies occurring in high‐gradient regions near the target edges.

**FIGURE 4:**
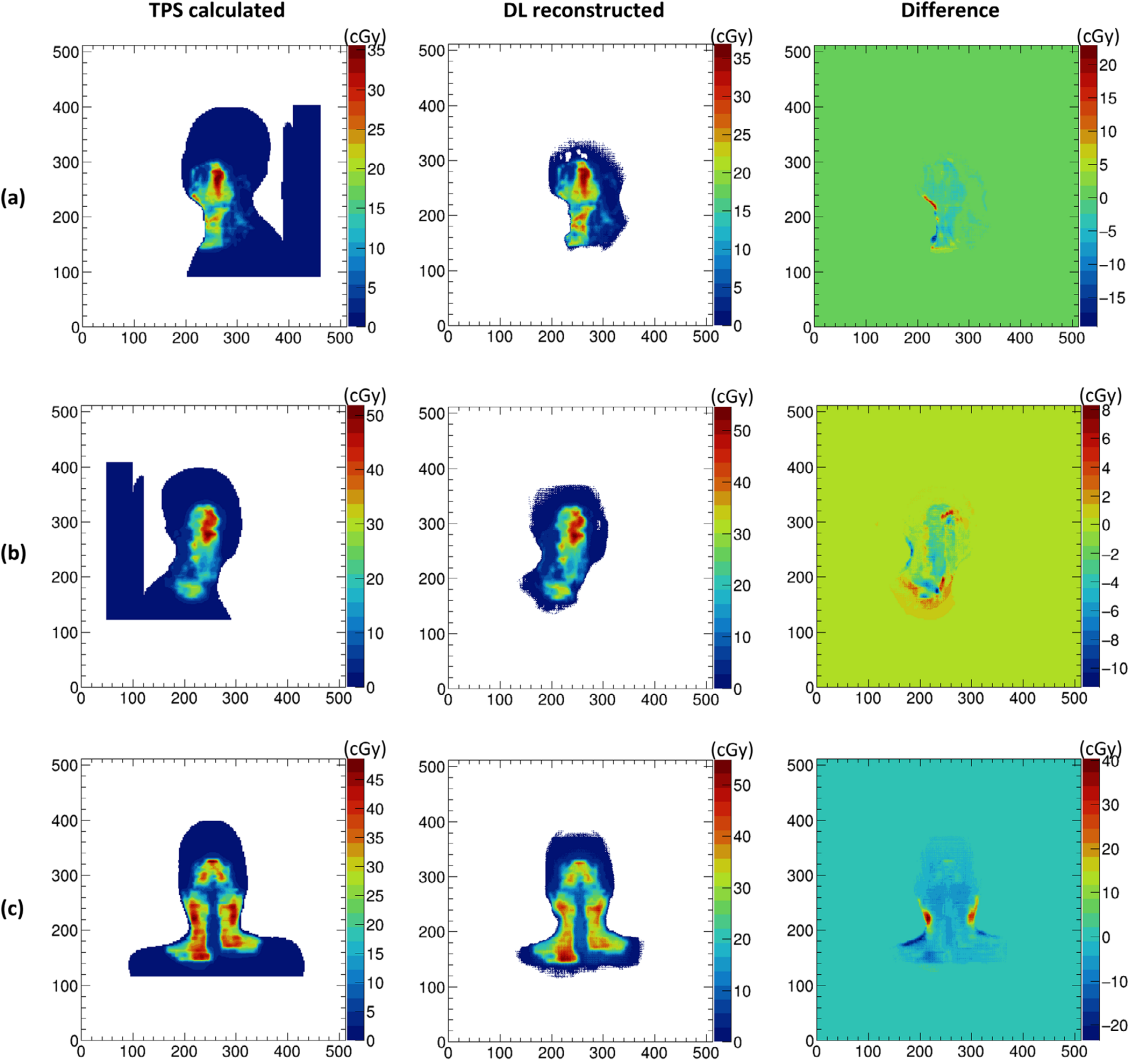
The overall dose distribution comparison in the transverse plane of TPS‐calculated (first column) and DL‐reconstructed (second column), the pixel‐wise dose difference between them (third column), relative dose errors in percent (forth column) and line profiles of red lines (last column) for three test cases, respectively in (a–c).

Figure 4 shows the cumulative dose distribution across all beams in the axial plane for three patients, along with pixel‐wise absolute and relative errors. The reconstructed dose maintained overall shape and gradient patterns, although localized underestimations were observed in high‐dose regions. This is further supported by line profiles and difference maps shown in the same figure.

**FIGURE 5:**
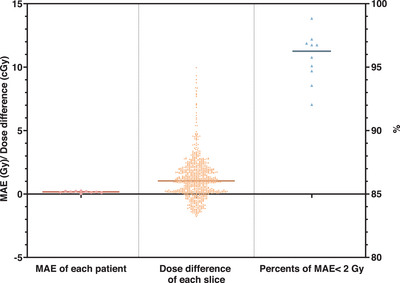
Statistical results of the dose difference, including the average MAE within the body contour of each test case (left column), the average dose difference per slice for all test cases (middle column), and the percentage of voxels within the body contour with MAE less than 2 for each case (right column).

Figure 5 summarizes voxel‐level accuracy across test cases. The MAE for total 3D dose within the body contour was less than 0.255 Gy in all cases (mean: 0.163 Gy). The average per‐slice dose difference across all cases was 1.186 Gy. More than 92% of voxels within the body exhibited a voxel‐wise MAE < 2 Gy, with a mean percentage of 95.76%, indicating reliable prediction in most anatomical regions.

In addition, global 3D gamma analysis yielded a passing rate of 90.67 % for the 3%/3 mm criterion. Table 1 summarizes the gamma passing rates from this study alongside those reported in previous deep learning‐based EPID dose reconstruction works.^38^, ^39^, ^40^

**TABLE 1:** Summary of previous deep learning–Based EPID dose reconstruction studies and the present work.

Study	Dataset size/ Site(s)	Delivery type	Model type	Gamma passing rate
Jia et al., 2021^38^	3 / Prostate, brain, and lung	IMRT	3DosiNet	98.6% (3%, 3 mm)
Martins et al., 2023^39^	16 / Pelvis	VMAT	3D U‐Net	>97% (3%, 2 mm)
Li et al., 2021^40^	18/ H&N, brain, lung, and rectum	IMRT	CNN	98.50% (3%, 2 mm)
Our study	10 / H&N	IMRT	2D Res‐UNet	90.67% (3%, 3 mm)

Across the 10 independent test patients, the patient‐level mean slice‐wise MAE showed a moderate positive correlation with the average modulation number (*r* = 0.82, *p* ≈ 0.006), indicating a tendency for higher‐MU plans to yield larger dose errors. Nonetheless, the relationship was not deterministic: for instance, Patient 2 exhibited both the highest modulation number (64.51) and a relatively high MAE (1.65 Gy), whereas Patient 6 with a similar modulation number (56.25) had a lower MAE (0.91 Gy), and Patient 5 with low modulation number (26.51) showed excellent accuracy (MAE 0.61 Gy). Full per‐patient modulation number/MAE summaries are provided in Table 2.

**TABLE 2:** Relationship between average modulation numbers and patient‐level mean slice‐wise MAEs across the 10 test patients (mean ± SD).

Patient	Modulation number	MAE
1	58.49 ± 15.61	1.98 ± 2.02
2	64.51 ± 23.26	1.65 ± 2.53
3	47.12 ± 10.39	0.94 ± 1.14
4	46.00 ± 14.59	1.41 ± 1.07
5	26.51 ± 5.42	0.61 ± 0.58
6	56.25 ± 17.88	0.91 ± 1.48
7	29.74 ± 7.42	0.74 ± 1.02
8	56.98 ± 19.55	1.51 ± 1.92
9	60.85 ± 16.92	1.18 ± 1.47
10	32.09 ± 7.59	0.75 ± 0.75

### 3.2 Dose‐volume histogram and clinical index comparison

**FIGURE 6:**
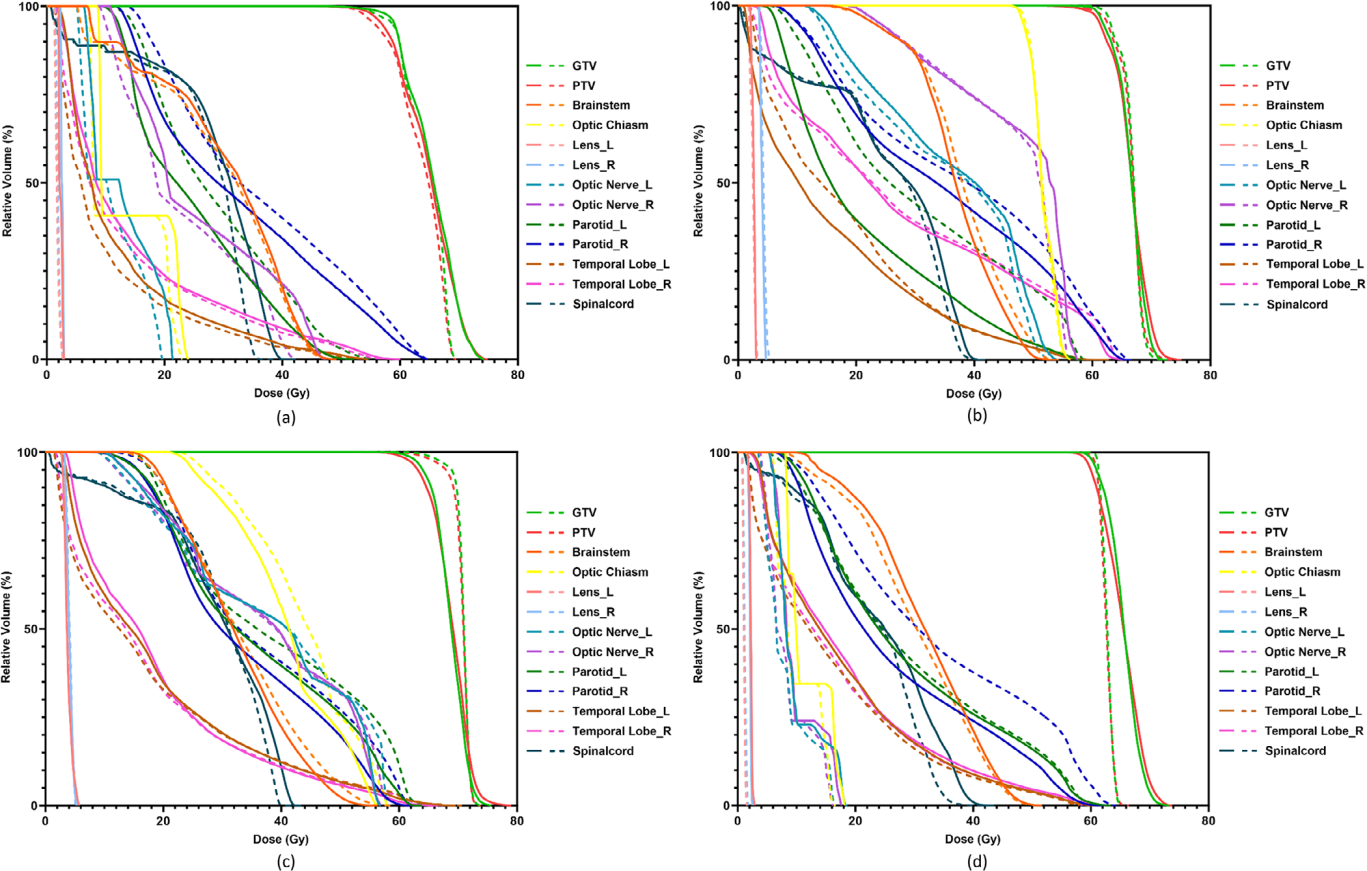
The examples of DVHs of DL reconstructed dose (solid line) and TPS calculated dose (dash line) for four test cases, respectively (a–d).

Figure 6 compares DVHs between the reconstructed and TPS‐calculated doses for four representative test cases. Overall, DVH curves for targets and OARs were well aligned between the two methods.

**FIGURE 7:**
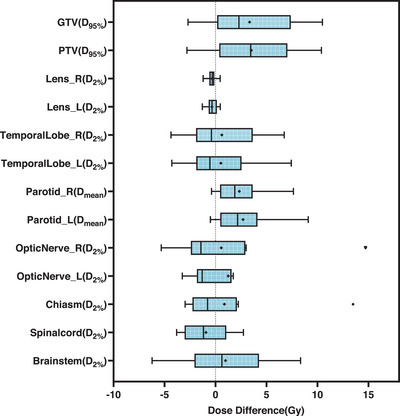
Box‐plots showing the DVH indices differences (TPS calculation minus DL reconstruction) for all test cases.

Quantitative differences in DVH indices across all test patients are presented in Figure 7 and Table 3. No statistically significant differences were observed in *D*_95%_ for GTV (*p* = 0.055) and PTV (*p* = 0.098), or in *D*_2%_ for the brainstem, spinal cord, chiasm, optic nerves, and temporal lobes (*p* > 0.05). Two statistically significant differences were identified: *D*_2%_ of the left lens (*p* = 0.020) and *D*_mean_ of the bilateral parotids (*p* = 0.012). These differences are attributable to low absolute doses in small‐volume structures (e.g., lens) and potential underestimation of dose near the body surface in parotid regions. Nevertheless, the absolute deviations in these indices remained within clinically acceptable limits, typically defined as < 3 Gy absolute dose deviation for lens and < 3% relative difference for other OARs.

**TABLE 3:** The DVH indices for 10 tested patients (mean ± SD).

Structure	DVH indices (Gy)	TPS calculated	DL reconstructed	*P*‐value (Wilcoxon signed‐rank test)
GTV	*D*_95%_	60.07 ± 12.8	56.74 ± 11.08	0.055
PTV	*D*_95%_	58.29 ± 12.83	54.90 ± 11.67	0.098
Lens_r	*D*_2%_	3.09 ± 1.95	3.42 ± 1.73	0.055
Lens_l	*D*_2%_	2.73 ± 1.82	3.18 ± 1.58	0.020*
Temporal lobe_R	*D*_2%_	49.45 ± 19.12	48.42 ± 17.46	0.652
Temporal lobe_L	*D*_2%_	46.41 ± 18.53	45.79 ± 16.46	>0.999
Parotid_R	*D*_mean_	32.26 ± 6.19	29.71 ± 6.33	0.012*
Parotid_L	*D*_mean_	25.93 ± 8.71	23.31 ± 8.14	0.012*
Optic nerve_R	*D*_2%_	36.97 ± 18.07	36.18 ± 16.79	0.910
Optic nerve_L	*D*_2%_	32.06 ± 19.35	30.51 ± 16.28	0.734
Chiasm	*D*_2%_	33.44 ± 18.81	32.15 ± 16.95	0.734
Spinal cord	*D*_2%_	35.74 ± 6.58	37.08 ± 7.19	0.074
Brainstem	*D*_2%_	45.76 ± 10.34	44.37 ± 9.14	0.496

*Note*: * indicates the relatively smaller Wilcoxon signed‐rank test results.

Abbreviation: *D*_2%_, the 2% volume coverage dose; *D*_95%_, the 95% volume coverage dose; *D*_mean_, the mean dose; GTV, gross tumor volume; PTV, planning target volume.

## 4 DISCUSSION

This study presents and evaluates a deep learning‐based framework for reconstructing three‐dimensional patient dose distributions directly from EPID images and planning CT without relying on traditional back‐projection or Monte Carlo methods. By employing a Res‐UNet architecture and mapping each beam's 2D portal image to depth‐dependent dose slices, the model enables efficient, per‐beam reconstruction of cumulative 3D dose in a manner compatible with clinical IMRT workflows.

The results demonstrate that the proposed method can reproduce overall dose patterns comparable to those calculated by the TPS, with voxel‐wise MAE below 0.255 Gy and more than 95% of voxels exhibiting MAE < 2 Gy. This level of accuracy, while not sufficient for adaptive re‐planning, is potentially suitable for real‐time treatment monitoring and patient‐specific QA. Importantly, the average per‐slice absolute error was 1.186 Gy—higher than the overall MAE—reflecting the fact that small cumulative deviations in individual high‐dose regions have a larger impact on slice‐based comparisons. These discrepancies were most prominent in areas with steep dose gradients, such as target margins or OAR interfaces, where local variations between adjacent slices are difficult to capture using a purely slice‐wise architecture. As shown in Figure 5, this underlines a limitation of the current model: it is prone to dose underestimation in high‐dose areas due to independent slice prediction without inter‐slice spatial continuity constraints.

In our cohort, cases with higher average modulation numbers tended to show larger errors as illustrated in Table 2. However, the effect size was modest and several counterexamples suggest that MUs/prescription dose alone is not a reliable indicator of plan complexity. This association may be influenced by stronger modulation, smaller field apertures, or steeper dose gradients, while anatomical variation and beam arrangement likely contribute as well. To more clearly identify these effects, future work should include additional plan complexity metrics (e.g., segment or control point counts, modulation complexity score, and aperture area or leaf travel) and use multivariable modeling on a larger dataset.

In comparison with previous DL‐based EPID dose reconstruction studies, the gamma passing rate in our study was slightly lower than previous studies, while still clinically acceptable (>90%). This difference may be explained by variations in datasets, the use of fully 3D network architectures in prior work, and their two‐stage coarse‐to‐refined strategy, which can enhance both global and local dose accuracy. The model's training process benefited from an architecture that implicitly learned depth‐dose relationships based on the inverse square law and anatomical context, without requiring interpolation or transformation of input features. This design contrasts with prior methods that relying interpolating planar dose estimates at discrete depths, or required coarse 3D training doses derived from computationally intensive simulations.^40^, ^43^ By directly predicting dose slices from native EPID images and CT inputs, the proposed approach simplifies the data pipeline and increases compatibility with clinical integration.

DVH analysis further supported the method's feasibility, with most target and OAR indices showing good agreement with TPS calculations. Statistically significant differences were observed only for *D*_2%_ of the left lens and *D*_mean_ of the parotid glands. These differences are likely attributable to the lens's small volume and low absolute dose—where small numerical errors can become statistically significant—and to underestimation of superficial dose near the skin surface in the parotid region. However, both discrepancies were small in absolute magnitude and clinically acceptable under standard tolerance levels.

Despite demonstrating the feasibility of EPID‐based in‐vivo dose reconstruction, several limitations remain. First, this study adopted a 2D Res‐UNet architecture due to hardware constraints. Although this design enabled training with limited GPU memory, it restricts the model's ability to incorporate inter‐slice contextual information. As a result, abrupt anatomical or dosimetric variations between adjacent slices—especially near high‐gradient regions—may not be accurately captured, leading to local underestimation and inter‐slice inconsistency. This effect is illustrated in Supplementary Figure 1, which shows the worst‐performing two slices from the test case with the lowest 3%/3 mm gamma passing rate. Future work will explore volumetric 3D models and transformer‐based architectures^44^, ^45^, ^46^ to better incorporate contextual information across slices and further improve prediction accuracy.

Second, the limited field of view of the EPID panel constrains the reconstruction of complete longitudinal dose distributions in extended treatment fields. For instance, inferior regions such as the supraclavicular lymph nodes in head and neck patients may lie partially outside the EPID projection range, leading to incomplete dose information at the field periphery and reduced reconstruction accuracy in those areas. In our test cohort, four cases exhibited peripheral truncation of EPID images, and in these patients the mean MAE of the inferior 10 slices was substantially higher compared with the remaining six cases (1.96 Gy vs. 0.55 Gy). Because the EPID field of view did not fully encompass these anatomical regions, the absence of projection data impaired the accuracy of dose prediction. Furthermore, this truncation effect was not explicitly accounted for during error analysis, which may have introduced a systematic bias into the overall MAEtes.

Third, the current framework is restricted to fixed‐beam IMRT plans, where one portal image per field allows for straightforward per‐angle reconstruction. This structure does not readily generalize to arc‐based techniques such as VMAT or tomo‐therapy, where continuous gantry motion produces cumulative EPID projections. Accurate modeling under such scenarios would require reformulating the data structure and possibly adopting new model architectures.

This study focused solely on head and neck cancer due to data availability and signal‐dose consistency. As an initial demonstration, the reconstructed dose results of one field for a single lung cancer case are provided in Supplementary Figure 2. Although the model was able to capture some features of the dose distribution, discrepancies in absolute dose values, particularly in high‐dose regions, were observed. These discrepancies highlight the complexities of generalizing the model across diverse anatomical sites. We will also focus on extending the model to additional anatomical sites, such as thoracic and pelvic regions, in order to build a generalized dose reconstruction framework applicable to multiple cancer types.

Although Monte Carlo‐simulated EPID images were used for model training to minimize confounding variables, this also limits the evaluation of real‐world performance. Future studies should incorporate measured EPID images from clinical treatments to assess robustness to inter‐fraction anatomical changes, setup errors, and delivery uncertainties. Integration of daily CT or cone‐beam CT data could further enhance adaptability for online dose monitoring and feedback control. Besides, although we did not apply intensity normalization to CT or EPID inputs in this study, we recognize that such normalization may improve cross‐device generalizability. Future work will explore normalization strategies and dual‐network architectures to better disentangle spatial and absolute dose learning.

The test cohort in our study comprised only 10 patients, which reduces the statistical power of the analysis. This constraint arose because, following a software update to the EPID simulation algorithm by UIH, the numerical characteristics of the simulated EPID signal changed substantially compared to earlier data. The updated simulations became incompatible with our pre‐update dataset and could not be integrated into the same evaluation pipeline without retraining the model from scratch. Although the sample size is consistent with several prior deep learning‐based dose prediction studies, the conclusions of this work should be regarded as exploratory, and further validation with larger and more diverse cohorts is necessary.

From a clinical perspective, the key advantage of this framework is its computational efficiency and workflow simplicity. Once trained, the model reconstructs per‐beam dose in under 15 s without requiring complex modeling or GPU acceleration, making it suitable for online applications. Although the current model does not achieve high‐precision reconstruction in all regions, it is capable of providing approximate, patient‐specific 3D dose estimates in real time, with the potential to trigger clinical alerts when deviations from the plan exceed tolerance thresholds.

In summary, our results indicate that deep learning‐based reconstruction of patient dose distributions from EPID and CT inputs is both achievable and clinically meaningful, particularly for streamlining in vivo verification in IMRT. Despite current limitations in high‐gradient areas and anatomical scope, this approach provides a valuable basis for future integration of AI‐driven QA tools in radiotherapy workflows.

## 5 CONCLUSION

This study demonstrates the feasibility of reconstructing 3D patient dose distributions directly from CT and EPID images using a Res‐UNet architecture, tailored for IMRT plans. The proposed method enables accurate and efficient dose estimation without complex physical modeling, showing good agreement with TPS‐calculated doses. These results support its potential for integration into online adaptive radiotherapy workflows, contributing to improved treatment verification and clinical workflow efficiency.

## AUTHOR CONTRIBUTIONS

All authors contributed to the study conception and design. Material preparation, data collection, and analysis were performed by Dong Yang and Jiawei Fan. The first draft of the manuscript was written by Dong Yang and all authors commented on previous versions of the manuscript. All authors read and approved the final manuscript.

## CONFLICT OF INTEREST STATEMENT

The authors declare no conflict of interest.

## FUNDING STATEMENT

This research was is sponsored by the Shanghai Pujiang Programme (No.23PJD014).

## ETHICAL STATEMENT

The Ethics Committee of Fudan University Shanghai Cancer Center approved the use of patient treatment plan samples in this study (approval number: 1612167‐18).

## Supporting information



Supplementary Figure 1. Dose difference maps and corresponding gamma analysis for the worst‐performing two slices from the test case with the lowest 3%/3 mm gamma passing rate.

Supplementary Figure 2. Reconstructed dose distribution of one field for a single lung cancer case using the early exploratory model.

## Data Availability

The datasets generated and analyzed during the current study are not publicly available due to privacy or ethical restrictions. However, data are available from the corresponding author upon reasonable request.

## References

[1] World Health Organization . Radiotherapy risk profile: technical manual. Geneva, Switzerland: World Health Organization, 2008.

[2] Graveling M , Williams M , Erridge S , et al. Towards safer radiotherapy. Imaging & Therapy Practice, 2008: 18.

[3] Derreumaux S , Etard C , Huet C , et al. Lessons from recent accidents in radiation therapy in France. Radiation Protection Dosimetry. 2008;131(1):130‐135. 18725379 10.1093/rpd/ncn235

[4] Hodapp N . The ICRU Report 83: prescribing, recording and reporting photon‐beam intensity‐modulated radiation therapy (IMRT). Strahlenther Onkol. 2012;188(1):97‐99. 22234506 10.1007/s00066-011-0015-x

[5] Olaciregui‐Ruiz I , Beddar S , Greer P , et al. In vivo dosimetry in external beam photon radiotherapy: requirements and future directions for research, development, and clinical practice. Phys Imaging Radiat Oncol. 2020;15:108‐116. 33458335 10.1016/j.phro.2020.08.003 PMC7807612

[6] Bailey DW , Kumaraswamy L , Bakhtiari M , Malhotra HK , Podgorsak MB . EPID dosimetry for pretreatment quality assurance with two commercial systems. J Appl Clin Med Phys. 2012;13(4):3736. 22766944 10.1120/jacmp.v13i4.3736 PMC5716510

[7] Huang YC , Yeh CY , Yeh JH , et al. Clinical practice and evaluation of electronic portal imaging device for VMAT quality assurance. Med Dosim. 2013;38(1):35‐41. 22854426 10.1016/j.meddos.2012.05.004

[8] Vazquez Quino LA , Chen X , Fitzpatrick M , et al. Patient specific pre‐treatment QA verification using an EPID approach. Technol Cancer Res Treat. 2014;13(1):1‐10. 23819492 10.7785/tcrt.2012.500351

[9] McDermott LN , Louwe RJ , Sonke JJ , van Herk MB , Mijnheer BJ . Dose‐response and ghosting effects of an amorphous silicon electronic portal imaging device. Med Phys. 2004;31(2):285‐295. 15000614 10.1118/1.1637969

[10] Zhang M , Qin S , Chen T , et al. A clinical objective IMRT QA method based on portal dosimetry and electronic portal imager device (EPID) measurement. Technol Cancer Res Treat. 2013;12(2):145‐150. 23289479 10.7785/tcrt.2012.500314

[11] Greer PB . Correction of pixel sensitivity variation and off‐axis response for amorphous silicon EPID dosimetry. Med Phys. 2005;32(12):3558‐3568. 16475754 10.1118/1.2128498

[12] Mijnheer BJ , González P , Olaciregui‐Ruiz I , Rozendaal RA , van Herk M , Mans A . Overview of 3‐year experience with large‐scale electronic portal imaging device‐based 3‐dimensional transit dosimetry. Pract Radiat Oncol. 2015;5(6):e679‐e687. 26421834 10.1016/j.prro.2015.07.001

[13] Celi S , Costa E , Wessels C , Mazal A , Fourquet A , Francois P . EPID based in vivo dosimetry system: clinical experience and results. J Appl Clin Med Phys. 2016;17(3):262‐276. 10.1120/jacmp.v17i3.6070 PMC5690938 27167283

[14] McCurdy BMC , McCowan PM . In vivo dosimetry for lung radiotherapy including SBRT. Phys Med. 2017;44:123‐130. 28576581 10.1016/j.ejmp.2017.05.065

[15] Esposito M , Piermattei A , Bresciani S , et al. Improving dose delivery accuracy with EPID in vivo dosimetry: results from a multicenter study. Strahlenther Onkol. 2021;197(7):633‐643. 33594471 10.1007/s00066-021-01749-6

[16] Chytyk‐Praznik K , VanUytven E , vanBeek et al. Model‐based prediction of portal dose images during patient treatment. Med Phys. 2013;40(3):031713. 23464308 10.1118/1.4792203

[17] Juste B , Miro R , Diez S , Campayo JM , Verdu G . Dosimetric capabilities of the Iview GT portal imager using MCNP5 Monte Carlo simulations. Annu Int Conf IEEE Eng Med Biol Soc. 2009;2009:3743‐3746. 19965234 10.1109/IEMBS.2009.5334899

[18] Yoon J , Jung JW , Kim JO , Yeo I . A Monte Carlo calculation model of electronic portal imaging device for transit dosimetry through heterogeneous media. Med Phys. 2016;43(5):2242. 27147336 10.1118/1.4945276

[19] Peca S , Sinha RS , Brown DW , Smith WL . In vivo portal imaging dosimetry identifies delivery errors in rectal cancer radiotherapy on the belly board device. Techno Cancer Res Treat. 2017;16(6):956‐963. 10.1177/1533034617711519 PMC5762054 28585490

[20] Francois P , Boissard P , Berger L , Mazal A . In vivo dose verification from back projection of a transit dose measurement on the central axis of photon beams. Phys Med. 2011;27(1):1‐10. 20615735 10.1016/j.ejmp.2010.06.002

[21] van Elmpt W , Nijsten S , Mijnheer B , Dekker A , Lambin P . The next step in patient‐specific QA: 3D dose verification of conformal and intensity‐modulated RT based on EPID dosimetry and Monte Carlo dose calculations. Radiother Oncol. 2008;86(1):86‐92. 18054102 10.1016/j.radonc.2007.11.007

[22] Parker BC , Shiu A , White RA , Maor M , Dong L , Liu HH . Pretreatment verification of IMSRT using electronic portal imaging and Monte Carlo calculations. Technol Cancer Res Treat. 2009;8(6):413‐423. 19925025 10.1177/153303460900800603

[23] Renner WD , Norton K , Holmes T . A method for deconvolution of integrated electronic portal images to obtain incident fluence for dose reconstruction. J Appl Clin Med Phys. 2005;6(4):22‐39. 10.1120/jacmp.v6i4.2104 PMC5723452 16421498

[24] McCurdy BM , Pistorius S . A two‐step algorithm for predicting portal dose images in arbitrary detectors. Med Phys. 2000;27(9):2109‐2116. 11011740 10.1118/1.1289375

[25] Wendling M , Louwe RJ , McDermott LN , Sonke JJ , van Herk M , Mijnheer BJ . Accurate two‐dimensional IMRT verification using a back‐projection EPID dosimetry method. Med Phys. 2006;33(2):259‐273. 16532930 10.1118/1.2147744

[26] Wendling M , McDermott LN , Mans A , et al. In aqua vivo EPID dosimetry. Med Phys. 2012;39(1):367‐377. 22225306 10.1118/1.3665709

[27] Sterckx B , Steinseifer I , Wendling M . *In vivo* dosimetry with an electronic portal imaging device for prostate cancer radiotherapy with an endorectal balloon. Phys Imaging Radiat Oncol. 2019;12:7‐9. Published 2019 Nov 9. 33458288 10.1016/j.phro.2019.10.002 PMC7807678

[28] van Elmpt WJ , Nijsten SM , Schiffeleers RF , et al. A Monte Carlo based three‐dimensional dose reconstruction method derived from portal dose images. Med Phys. 2006;33(7):2426‐2434. 16898445 10.1118/1.2207315

[29] McCowan PM , Asuni G , van Beek T , van Uytven E , Kujanpaa K , McCurdy BM . A model‐based 3D patient‐specific pre‐treatment QA method for VMAT using the EPID. Phys Med Biol. 2017;62(4):1600‐1612. 28079525 10.1088/1361-6560/aa590a

[30] Olaciregui‐Ruiz I , Rozendaal R , van Oers RFM , Mijnheer B , Mans A . Virtual patient 3D dose reconstruction using in‐air EPID measurements and a back‐projection algorithm for IMRT and VMAT treatments. Phys Med. 2017;37:49‐57. 28535915 10.1016/j.ejmp.2017.04.016

[31] McDermott LN , Wendling M , Nijkamp J , et al. 3D in vivo dose verification of entire hypo‐fractionated IMRT treatments using an EPID and cone‐beam CT. Radiother Oncol. 2008;86(1):35‐42. 18061692 10.1016/j.radonc.2007.11.010

[32] Alhazmi A , Gianoli C , Neppl S , et al. A novel approach to EPID‐based 3D volumetric dosimetry for IMRT and VMAT QA. Phys Med Biol. 2018;63(11):115002. Published 2018 May 22. 29714714 10.1088/1361-6560/aac1a6

[33] van Elmpt W , McDermott L , Nijsten S , Wendling M , Lambin P , Mijnheer B . A literature review of electronic portal imaging for radiotherapy dosimetry. Radiother Oncol. 2008;88(3):289‐309. 18706727 10.1016/j.radonc.2008.07.008

[34] Valdes G , Chan MF , Lim SB , Scheuermann R , Deasy JO , Solberg TD . IMRT QA using machine learning: a multi‐institutional validation. J Appl Clin Med Phys. 2017;18(5):279‐284. 28815994 10.1002/acm2.12161 PMC5874948

[35] Tomori S , Kadoya N , Takayama Y , et al. A deep learning‐based prediction model for gamma evaluation in patient‐specific quality assurance. Med Phys. Published online July 31, 2018. 10.1002/mp.13112 30066388

[36] Sakai M , Nakano H , Kawahara D , et al. Detecting MLC modeling errors using radiomics‐based machine learning in patient‐specific QA with an EPID for intensity‐modulated radiation therapy. Med Phys. 2021;48(3):991‐1002. 33382467 10.1002/mp.14699

[37] Kimura Y , Kadoya N , Tomori S , Oku Y , Jingu K . Error detection using a convolutional neural network with dose difference maps in patient‐specific quality assurance for volumetric modulated arc therapy. Phys Med. 2020;73:57‐64. 32330812 10.1016/j.ejmp.2020.03.022

[38] Jia M , Wu Y , Yang Y , et al. Deep learning‐enabled EPID‐based 3D dosimetry for dose verification of step‐and‐shoot radiotherapy. Med Phys. 2021;48(11):6810‐6819. 34519365 10.1002/mp.15218

[39] Martins JC , Maier J , Gianoli C , et al. Towards real‐time EPID‐based 3D in vivo dosimetry for IMRT with Deep Neural Networks: a feasibility study. Phys Med. 2023;114:103148. 37801811 10.1016/j.ejmp.2023.103148

[40] Li Y , Xiao F , Liu B , et al. Deep learning‐based 3D in vivo dose reconstruction with an electronic portal imaging device for magnetic resonance‐linear accelerators: a proof of concept study. Phys Med Biol. 2021;66(23). 10.1088/1361-6560/ac3b66 34798623

[41] Feng B , Yu L , Mo E , et al. Evaluation of daily CT for EPID‐based transit in vivo dosimetry. Front Oncol. 2021;11:782263. Published 2021 Nov 2. 34796120 10.3389/fonc.2021.782263 PMC8592931

[42] Chen L , Zhang Z , Yu L , et al. A clinically relevant online patient QA solution with daily CT scans and EPID‐based in vivo dosimetry: a feasibility study on rectal cancer. Phys Med Biol. 2022;67(22). 10.1088/1361-6560/ac9950 36220015

[43] Ma J , Nguyen D , Bai T , et al. A feasibility study on deep learning‐based individualized 3D dose distribution prediction. Med Phys. 2021;48(8):4438‐4447. 34091925 10.1002/mp.15025 PMC8842508

[44] Jiao Z , Peng X , Wang Y , et al. TransDose: transformer‐based radiotherapy dose prediction from CT images guided by super‐pixel‐level GCN classification. Med Image Anal. 2023 Oct;89:102902. 37482033 10.1016/j.media.2023.102902

[45] Gheshlaghi T , Nabavi S , Shirzadikia S , Moghaddam ME , Rostampour N . A cascade transformer‐based model for 3D dose distribution prediction in head and neck cancer radiotherapy. Phys Med Biol. 2024 Feb 5;69(4):045010. 10.1088/1361-6560/ad209a 38241717

[46] Ahn SH , Kim ES , Kim C , et al. Deep learning method for prediction of patient‐specific dose distribution in breast cancer. Radiat Oncol. 2021;16(1):154. 34404441 10.1186/s13014-021-01864-9 PMC8369791

