# Supplementary material for: Deep learning‐based 3D dose reconstruction for intensity modulated radiation therapy using electronic portal imaging devices
Source: J Appl Clin Med Phys. 2025 Nov 5;26(11):e70328. doi: 10.1002/acm2.70328 (PMC12589810; doi:10.1002/acm2.70328)
Supplement: Supplementary file 1 — Supplementary Figure 1. Dose difference maps and corresponding gamma analysis for the worst‐performing two slices from the test case with the lowest 3%/3 mm gamma passing rate. [file ACM2-26-e70328-s001.pdf]

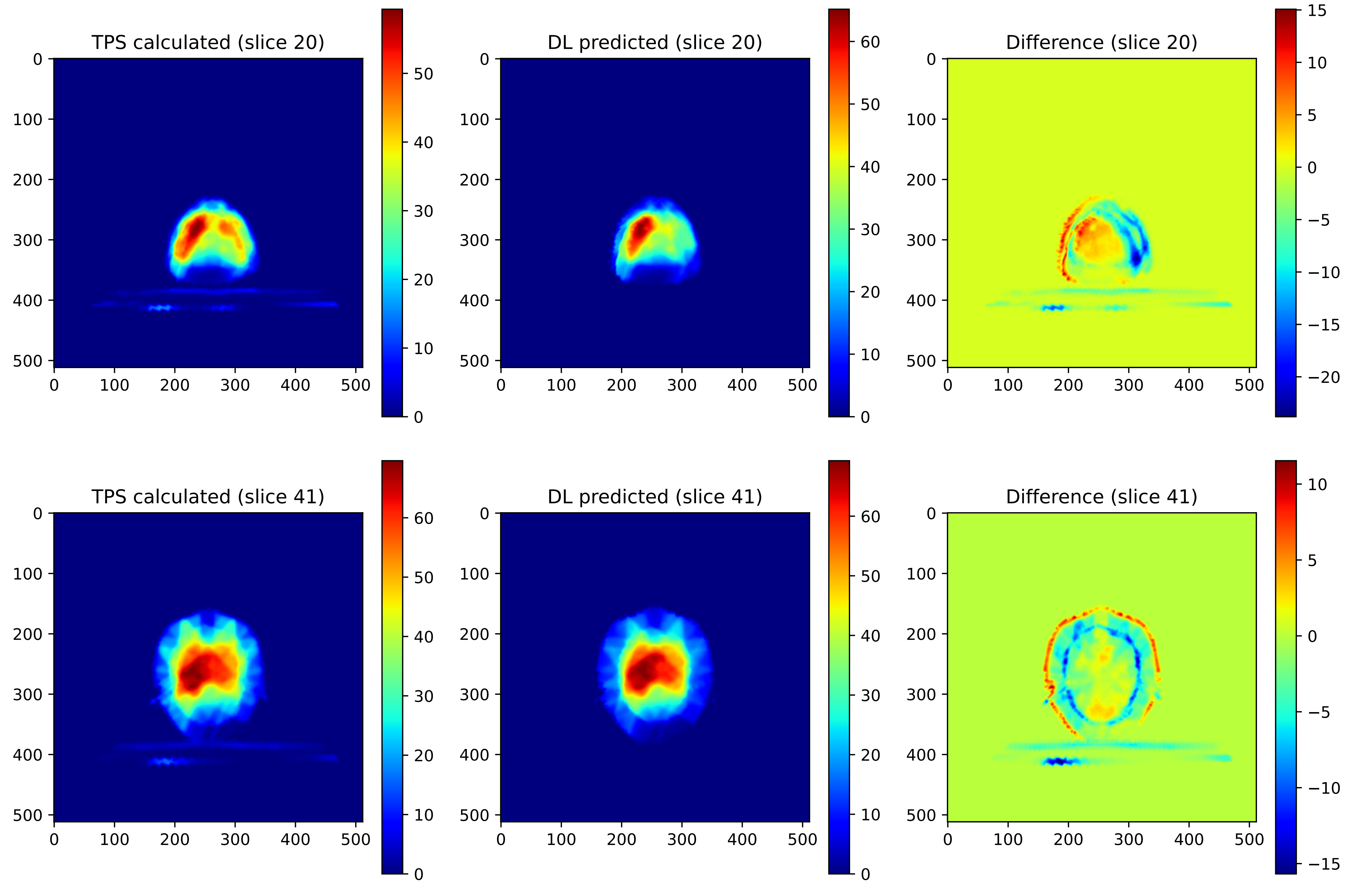

TPS calculated (slice 20)
DL predicted (slice 20)
Difference (slice 20)
TPS calculated (slice 41)
DL predicted (slice 41)
Difference (slice 41)
