# Supplementary figures and images for: Deep learning‐based 3D dose reconstruction for intensity modulated radiation therapy using electronic portal imaging devices
Source: J Appl Clin Med Phys. 2025 Nov 5;26(11):e70328. doi: 10.1002/acm2.70328 (PMC12589810; doi:10.1002/acm2.70328)

TPS calculated

DL reconstructed

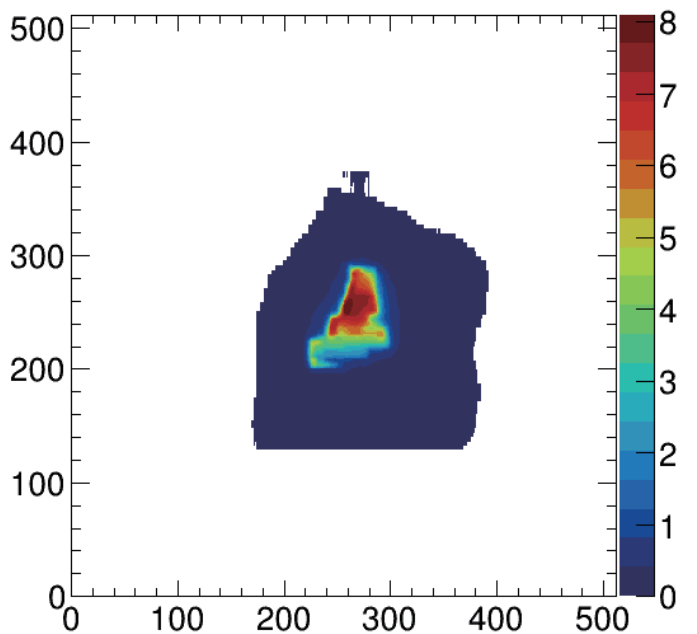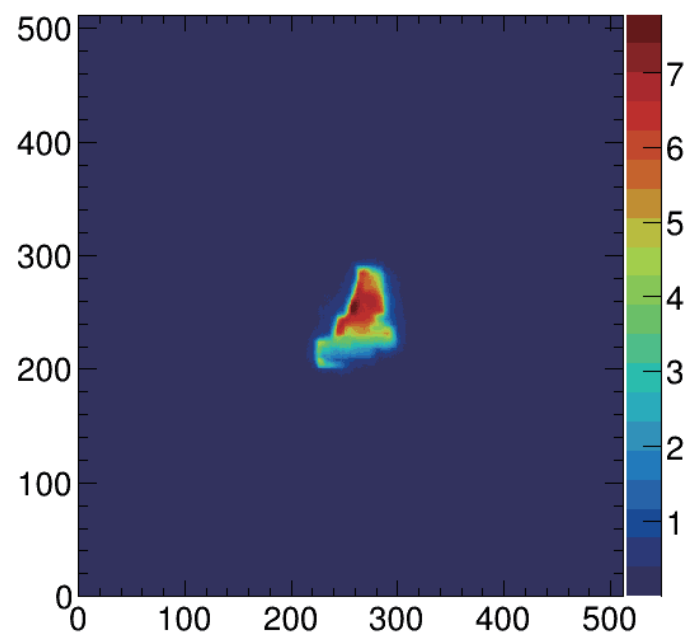

(a)

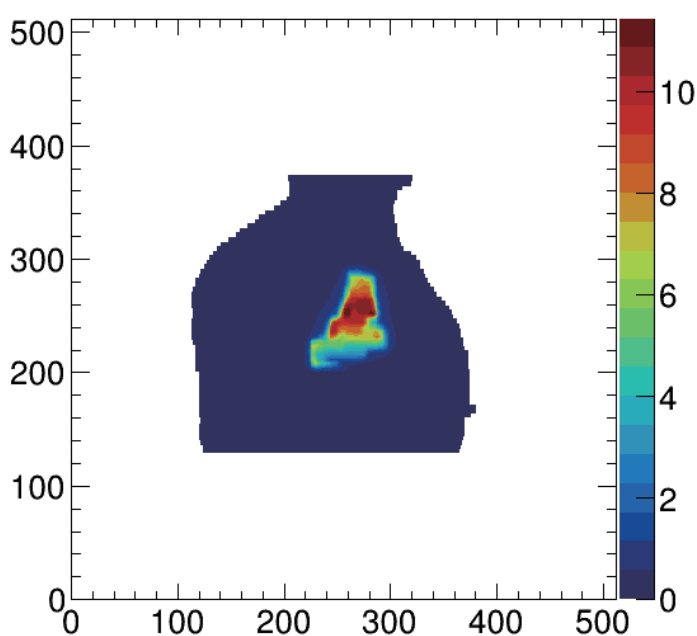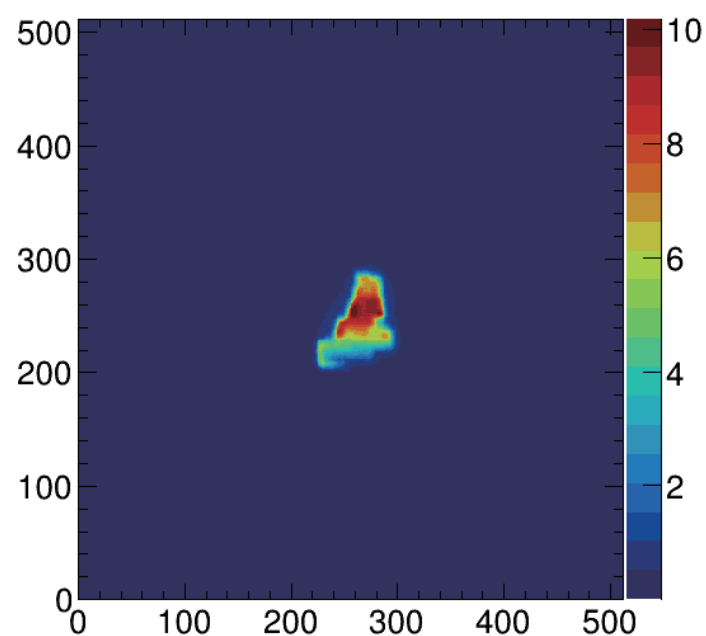

(b)

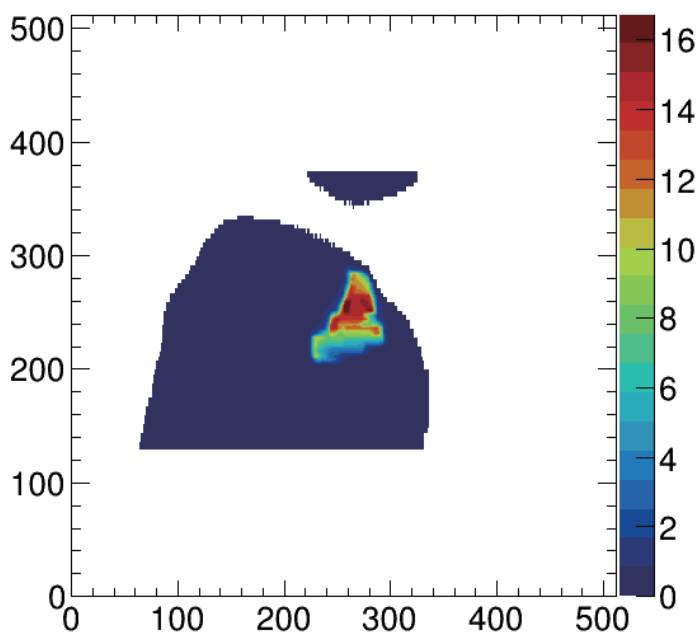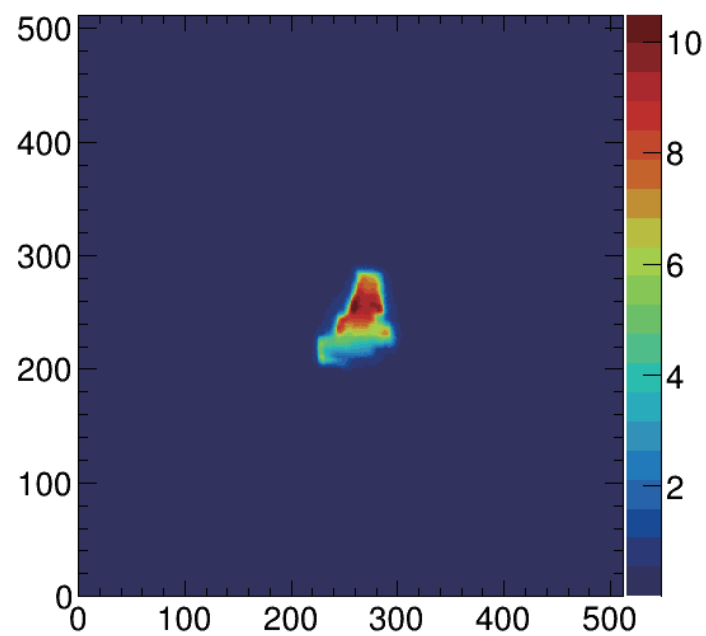

(c)

Supplement: Supplementary file 2 — Supplementary Figure 2. Reconstructed dose distribution of one field for a single lung cancer case using the early exploratory model. [file ACM2-26-e70328-s002.pdf]
